# Immunoguided cutaneous regeneration: a theoretical framework linking extracellular matrix, immunity, and skin allografts

**DOI:** 10.3389/fimmu.2026.1910184

**Published:** 2026-08-13

**Authors:** Marcelo Fonseca

**Affiliations:** 1 Department of Surgery, Universitat Autònoma de Barcelona, Barcelona, Spain; 2 Tarapacá Skin and Tissue Bank, Iquique, Chile

**Keywords:** constructive remodeling, extracellular matrix, immunoguided cutaneous regeneration, macrophage polarization, regenerative medicine, skin allograft, tissue regeneration, transplant immunology

## Abstract

Adult human skin healing is predominantly characterized by fibrosis rather than true regeneration, despite the conserved regenerative principles observed in fetal wound healing, liver regeneration, and other biological systems. Historical and contemporary observations in skin allotransplantation, including studies describing the differential behavior of the epidermis and dermis and recent experience with Cryopreserved Total Skin Allografts (CTSA), reveal a recurring biological pattern in which the epidermis is progressively eliminated while the dermal extracellular matrix remains relatively preserved. These observations suggest that immune-mediated tissue remodeling and regeneration may coexist during graft development. Here, we propose Immunoguided Cutaneous Regeneration (ICR) as a biologically plausible and experimentally testable conceptual framework to explain these observations. ICR proposes that regeneration associated with certain skin allografts depends not on prolonged donor cell survival but rather on the convergence of a preserved dermal extracellular matrix and a regulated alloimmune response, neither of which is proposed to be sufficient in isolation. Within this framework, the graft is proposed to function primarily as a transient instructive microenvironment, guiding recipient cell recruitment, matrix remodeling, and tissue reorganization. The process is organized into four partially overlapping phases: biological barrier, immune activation and cellular colonization, selective epidermal elimination, and regenerative remodeling. Therefore, regeneration is interpreted as a context-dependent emergent property of the matrix–host system, arising when a preserved extracellular matrix and a regulated, resolving alloimmune response converge within a permissive biological environment. ICR integrates previously fragmented historical, experimental, and clinical observations into a coherent mechanistic model that bridges the fields of transplantation immunology, extracellular matrix biology, and regenerative medicine. Although the available evidence does not establish causality, it is consistent with the proposed framework and supports its biological plausibility of the framework. Importantly, the theory generates explicit and potentially falsifiable predictions that distinguish it from alternative explanations and provide a structured conceptual foundation for future experimental investigations. If validated, ICR may redefine skin allotransplantation not only as a model of immune rejection but also as a model of context-dependent tissue regeneration, providing a conceptual basis for understanding how immunity, extracellular matrix biology, and tissue architecture collectively determine the balance between fibrosis and regeneration.

## 1 Introduction

Since Aristotle described in Historia Animalium the capacity of lizards to regenerate their tails following amputation (1), the reconstruction of complete body parts in organisms such as salamanders and zebrafish has remained one of the most enigmatic phenomena in biology (2–4). In contrast, mammals respond to tissue damage predominantly through repair and fibrosis, generating scars that restore anatomical continuity but do not reproduce the structure or function of the original tissues (5). Despite this divergence, organisms with high regenerative capacity share a set of surprisingly conserved biological principles: a protective barrier, a regulated innate immune response, an instructive extracellular matrix, coordinated recruitment of reparative cells, and transient reactivation of developmental programs (6–15).

These elements do not represent independent phenomena; instead, they constitute an integrated biological sequence consistently observed across multiple regenerative models (16). In this context, the immune response occupies a central position, coordinating the interaction between the extracellular matrix, resident cells, and recruited cells during the regenerative process while actively participating in angiogenesis and tissue remodeling (17, 18). Disruption or interruption of any of these components typically results in regenerative failure and replacement of the lost tissue by fibrosis or scar formation (19, 20), suggesting that regeneration depends less on an isolated mechanism and more on the coordinated convergence of these elements.

However, regeneration is not entirely absent in mammals or humans. Phenomena such as scarless fetal wound healing and liver regeneration following partial hepatectomy demonstrate that under specific biological conditions, human tissues retain a significant capacity to restore their structure and function (21, 22). These observations suggest that regeneration is not an exclusive property of particular species but rather a biological phenomenon that emerges when mechanisms capable of generating a regenerative microenvironment converge (10).

In recent decades, regenerative medicine has sought to identify these mechanisms and reproduce them therapeutically using strategies based on cells, growth factors, biomaterials, and tissue engineering (23, 24). However, attention has progressively shifted from individual components to the microenvironment that regulates their behavior and determines their biological fate (12). This conceptual shift has positioned the extracellular matrix and immune response as principal candidates for explaining how regeneration emerges across diverse biological systems (11, 17).

Understanding how these elements interact to promote regeneration or, conversely, lead to fibrosis, constitutes one of the major challenges in contemporary regenerative medicine. In this context, the growing body of evidence linking the extracellular matrix to immune regulation, cellular recruitment, and tissue remodeling suggests that regeneration may depend not only on matrix architecture but also on the manner in which this architecture modulates and reorganizes the host response (25, 26). This interaction provides an opportunity to reinterpret biological phenomena that were previously considered independent and to explore new conceptual frameworks for understanding tissue reconstruction.

In this study, we propose the concept of Immunoguided Cutaneous Regeneration (ICR), a theoretical framework suggesting that the coordinated interaction between a preserved extracellular matrix and a regulated immune response can generate a microenvironment capable of promoting dermal reconstruction. Based on historical observations from skin allotransplantation, evidence from regenerative biology, and contemporary clinical observations, we propose that phenomena traditionally interpreted as manifestations of rejection may instead represent stages of a more complex regenerative process, in which the extracellular matrix of the graft, functionally understood as a matrix transplant rather than as transferred tissue, acts as a biologically active element capable of instructing tissue reconstruction through the transient generation of an immunoregenerative microenvironment (16, 27, 28). To develop this hypothesis, we reviewed the conceptual evolution of regenerative medicine, the instructive role of the extracellular matrix, examples of regeneration observed in humans, and historical evidence supporting the biological reinterpretation of skin graft rejection. Finally, we propose biological mechanisms that may underpin this model and formulate a set of testable predictions to evaluate its experimental and clinical validity.

## 2 The evolution of regenerative medicine

The development of regenerative medicine has been marked by a progressive shift in the biological targets considered essential for tissue restoration. Early approaches focused primarily on cells under the premise that regeneration could be achieved through the replacement of cell populations lost after injury. This view drove the development of cell therapies and stem cell-based strategies aimed at reconstructing damaged tissues and organs through the direct delivery of cells with regenerative potential (24).

However, over time, it became evident that cells alone were insufficient to explain the complexity of regenerative processes observed in nature. Attention has shifted toward growth factors and other signaling molecules that regulate cell proliferation, migration, differentiation, and survival. This new approach has led to the development of multiple therapeutic strategies designed to stimulate regeneration through the administration of biological signals that can activate specific reparative programs (24).

The integration of cells, molecular signals, and structural support has led to the development of tissue engineering. This paradigm, classically known as the tissue engineering triad, proposes that tissue reconstruction requires the coordinated interaction of three fundamental components: cells, regulatory factors, and a scaffold capable of providing spatial organization and mechanical support (23). Consequently, a wide variety of natural and synthetic biomaterials have been developed to promote cellular colonization and new tissue formation.

Despite these advances, many developed biomaterials have failed to fully reproduce the outcomes observed in naturally regenerative systems. This limitation prompted a deeper reconsideration of the mechanisms governing regeneration. Numerous studies have demonstrated that identical cellular populations can exhibit radically different behaviors depending on the biological environment in which they are located. Consequently, the focus of regenerative medicine has progressively shifted from cells and biomaterials to the microenvironment that regulates their behavior (12).

Within this conceptual evolution, the extracellular matrix has been recognized as a central regulator of tissue homeostasis, rather than a passive element solely responsible for maintaining tissue architecture (11). In parallel, the immune system has ceased to be viewed exclusively as a mediator of inflammation and tissue damage and has begun to be understood as an active participant in regeneration and tissue remodeling (17, 18). These observations have led to an increasingly accepted concept that regeneration depends not only on the presence of cells with regenerative potential but also on a microenvironment capable of coordinating the cellular, immunological, and molecular responses required to reconstruct injured tissue.

In this context, the extracellular matrix emerges as the principal integrator of these signals, transforming the classical view of the biological scaffold into a system capable of storing, transmitting, and organizing information required for tissue reconstruction.

## 3 The extracellular matrix is not a passive scaffold

For much of the twentieth century, the extracellular matrix (ECM) was viewed primarily as a structural element responsible for providing mechanical support and physical organization to the tissues. From this perspective, cells are regarded as the true functional units of the organism, whereas the matrix occupies a secondary role, limited to the maintenance of tissue architecture. However, advances in developmental biology, cell biology, and regenerative medicine have profoundly transformed this field.

One of the earliest fundamental contributions came from Paul Weiss, who proposed that cellular behavior cannot be understood in isolation and is conditioned by the structural context surrounding the cells. His studies have demonstrated that cells are not randomly distributed within tissues but rather respond to physical signals present in their environment, organizing their migration, orientation, and behavior according to tissue architecture. These observations anticipated concepts that later became the central pillars of regenerative biology (29, 30).

Subsequently, Grobstein demonstrated that morphogenetic information could be transmitted through acellular matrices interposed between different cellular populations. These experiments provided some of the first evidence that the extracellular matrix could function as an active carrier of biological signals rather than merely as an inert support structure (31).

This view was consolidated by Bissell through the concept of dynamic reciprocity, according to which cells continuously modify their extracellular matrix, while simultaneously, the matrix regulates gene expression, differentiation, and cellular behavior. From this perspective, the signals that determine tissue organization do not reside exclusively within the cellular genome but also within the structural, biochemical, and mechanical components of the extracellular microenvironment (32, 33).

Recently, Badylak and other investigators demonstrated that biological extracellular matrices possess a remarkable capacity to modulate the immune response, promote angiogenesis, and facilitate constructive remodeling processes. In various experimental and clinical models, extracellular matrices have been shown to induce tissue responses that extend far beyond the functions expected of a physical support structure, suggesting the existence of instructive properties capable of actively influencing the fate of injured tissues (34, 35).

It is now recognized that the extracellular matrix transmits signals not only through its static composition but also through dynamic remodeling processes. On the one hand, the interaction between cells and the matrix through specialized receptors, particularly integrins, enables the translation of structural and mechanical signals into biological responses capable of regulating cell migration, proliferation, differentiation, and survival (20). In contrast, the physiological degradation of the matrix by metalloproteinases and other proteases releases growth factors previously sequestered within it, including TGF-β, fibroblast growth factors, insulin-like growth factors, and Wnt ligands, generating signaling gradients capable of modifying the behavior of resident and recruited cells (20).

In a complementary manner, matrix remodeling can reveal previously concealed biological signals through two related mechanisms. Proteolytic degradation generates bioactive fragments derived from structural proteins, known as matricryptins, which regulate angiogenesis, inflammation, and tissue repair processes. In addition, mechanical deformation or structural reorganization of the matrix can expose cryptic sites normally hidden within proteins, such as fibronectin, thereby revealing new biological signals that are inaccessible in an intact matrix. These mechanisms transform the extracellular matrix into a dynamic system capable of generating and reorganizing biological signals according to the needs of the tissue (11).

Consequently, the extracellular matrix not only regulates the behavior of resident cells but also actively participates in the recruitment (homing) of recipient-derived cells. Cellular migration observed during repair and regeneration depends largely on matrix-derived signals, which direct cellular trafficking toward specific areas of injury and contribute to the spatial organization of the regenerative response (13).

Taken together, these observations have led to a substantial reinterpretation of the role of the extracellular matrix in tissue biology. Rather than a passive scaffold, the matrix can be understood as a biological information system capable of storing, integrating, releasing, and transmitting signals that guide cellular behavior. From this perspective, regeneration is not merely the result of the activity of individual cells but rather an emergent property arising from the dynamic interaction between recipient-derived cells, the immune response, and the signals contained within the extracellular microenvironment surrounding them. This concept provides the biological foundation necessary to understand how certain preserved matrices may actively participate in tissue reconstruction and constitutes the conceptual basis upon which the Immunoguided Cutaneous Regeneration model proposed in this study is built.

## 4 Human regeneration: lessons from fetal wound healing and liver regeneration

If the extracellular matrix is a central component of the regenerative microenvironment, it is reasonable to question whether the regenerative phenomena observed in humans are dependent on the tissue context. The predominantly fibrotic response in mammals does not imply that humans lack regenerative competence; rather, this capacity remains largely latent and is subject to specific biological conditions that inhibit its expression. Two paradigmatic examples illustrate this principle: scarless fetal wound healing and liver regeneration following partial hepatectomy. Although they involve different tissues and biological contexts, both share an essential characteristic: regeneration occurs when the microenvironment, particularly the extracellular matrix and immune response, adopts a permissive configuration. Taken together, these models suggest that human regenerative capacity has not been lost but remains constrained by the biological conditions surrounding injury response.

Fetal wound healing is one of the most extensively studied examples of tissue regeneration in mammalian species. During the early stages of embryonic development, cutaneous wounds can heal without scar formation, restoring the original dermal architecture and specialized structures, such as skin appendages. This capacity is associated with a distinctive microenvironment characterized by an extracellular matrix rich in hyaluronic acid and type III collagen, which is composed of fine and highly organized fibers and a markedly attenuated inflammatory response. Fetal wounds exhibit reduced recruitment and activation of neutrophils and macrophages (36, 37), along with a cytokine profile dominated by regeneration-associated signals, including a predominance of TGF-β3 over profibrotic isoforms TGF-β1 and TGF-β2 (38). Particularly revealing is the fact that the transition from regenerative to fibrotic healing occurs at a defined stage of fetal development and coincides with the progressive maturation of the immune response. This observation suggests that scar formation is not an inevitable outcome of tissue repair but rather a consequence of the immunological and matrix contexts in which repair occurs (21, 36).

The second example is liver regeneration. Following the resection of a substantial portion of its mass, the liver can restore its size and function through a highly coordinated response involving hepatocytes, non-parenchymal cells, immune mediators, and extracellular matrix remodeling. Although this process differs from the epimorphic regeneration observed in organisms such as salamanders and zebrafish, it shares a fundamental characteristic with them: regeneration emerges from a dynamic interaction between immunity and the microenvironment of the organism. As extensively reviewed by Michalopoulos and Bhushan, the innate immune response plays an essential initiating role, in which Kupffer cells and mediators such as TNF-α and IL-6 prime hepatocytes to enter proliferation, while controlled remodeling of the extracellular matrix releases growth factors previously sequestered within it, among which hepatocyte growth factor (HGF) is one of the principal drivers of liver regeneration (22). Recent studies have expanded this view by demonstrating that the hepatic extracellular matrix functions as a dynamic niche capable of serving as a reservoir for growth factors, transmitting mechanical signals, and coordinating communication among multiple cellular populations during regeneration. In this context, the extracellular matrix and immune system do not act as passive bystanders but as active organizers of the regenerative response, coordinating the events required to restore the functional mass of the organ (39, 40).

Despite their anatomical and physiological differences, fetal wound healing and liver regeneration converge on common biological pathways. In both cases, regeneration is associated with the establishment of a microenvironment that limits disorganized inflammation, promotes controlled extracellular matrix remodeling, facilitates cellular recruitment (homing) and coordination, and allows transient activation of molecular programs that are normally associated with embryonic development. These observations reinforce the idea that regeneration does not depend exclusively on the species or organ involved but rather on the capacity to establish a biological environment capable of integrating the immunological, cellular, and matrix-derived responses required for the functional reconstruction of tissue (21, 22).

This convergence reframes the central question: the issue is not whether human tissues can regenerate, but under what conditions this latent program is permitted to be expressed. If regeneration depends on the convergence of a preserved instructive matrix and a regulated immune response, it is reasonable to ask whether a context capable of providing both conditions can reactivate such a program in the skin of adults. This possibility emerges in an unexpected and, at the same time, paradoxical manner in skin allotransplantation.

## 5 Skin allotransplantation: a biological paradox

If successful tissue regeneration depends not on the absence of inflammation but on the emergence of a microenvironment capable of coordinating immune responses, extracellular matrix remodeling, and cellular behavior, an important question arises: can such conditions develop transiently in adult tissues that would otherwise heal by fibrosis? Unexpectedly, skin allotransplantation may represent such a biological setting. Although historically regarded as the paradigm of immunological rejection, the studies that established this concept also reported observations that remain difficult to reconcile with a purely destructive view of the host response.

This paradox originates from seminal studies that laid the foundations of modern transplant immunology. In their classic work, *The Fate of Skin Homografts in Man*, Thomas Gibson and Peter B. Medawar demonstrated that skin homografts were ultimately destroyed by a specific host immune response, findings that later contributed to Medawar receiving the 1960 Nobel Prize in Physiology or Medicine for the discovery of acquired immunological tolerance. However, these studies also contained observations suggesting that different structural compartments of the skin may follow distinct biological trajectories during rejection, an aspect that has received comparatively little attention and provides an important conceptual foundation for the present hypothesis (41).

Several decades later, Yang Chih-Chun and colleagues described one of the most relevant clinical observations in this field while studying extensively burned patients treated with combinations of auto- and allogeneic skin grafts. The authors reported the so-called *sandwich phenomenon*, in which the autologous epidermis, expanding from islands of autograft, progressively replaced the rejected allogeneic epidermis, while the allogeneic dermis remained integrated within the recipient wound bed. Consequently, a functional allogeneic dermis persisted beneath the intact autologous epidermis. This observation was particularly significant because it demonstrated that rejection of the epidermis did not necessarily imply the simultaneous elimination of all graft components and suggested that the dermis could continue to participate in tissue repair, even in the presence of an active immune response (42).

In a complementary line of investigation, Cuono et al. demonstrated that following the initial engraftment of cryopreserved skin allografts in burn patients, the removal of the allogeneic epidermis allowed the remaining dermis to be used as a substrate for cultured autologous keratinocyte grafting. In these models, reconstruction of a functional dermoepidermal junction and structural integration between an autologous epidermis and the underlying allogeneic dermis were documented, resulting in a cutaneous architecture that approached normal. These findings reinforce the concept that the allogeneic dermis can function as a biologically competent substrate for cutaneous reconstruction rather than simply as a tissue destined for elimination (43, 44).

Cryopreserved Total Skin Allografts (CTSA) may be understood as a conceptual evolution of these historical models. Subjected to cryopreservation and terminal sterilization by irradiation, unlike traditional split-thickness allografts, they preserve the full thickness of the dermis, together with the three-dimensional architecture of its extracellular matrix. During clinical application, a consistent biological pattern has been observed, characterized by complete initial engraftment followed by the progressive appearance of a superficial epidermal eschar, while the underlying dermis shows signs of vascularization, cellular infiltration, and tissue remodeling. In some cases, this process culminates in the formation of a vascularized neodermis capable of serving as a platform for subsequent autografting or supporting spontaneous cutaneous regeneration. A potentially important difference between CTSAs and the allografts described in most historical literature is the preservation of the dermis in its full-thickness form. Conventional skin allografts used in burn care are predominantly split-thickness grafts harvested with a dermatome and, therefore, contain only a fraction of the native dermal architecture needed for optimal healing. In contrast, CTSAs preserve both the papillary and reticular dermis, maintaining a greater proportion of the extracellular matrix, its three-dimensional organization, and compositional gradients. From an ICR perspective, this difference may be biologically significant. Historically described regenerative phenomena may become more evident in CTSAs because they provide a more complete extracellular matrix scaffold, potentially preserving a greater amount of structural and biochemical information required to guide tissue remodeling and regeneration (27, 28).

These observations can be interpreted as independent phenomena when considered individually. However, when analyzed collectively, they reveal a remarkably consistent pattern extending from the work of Gibson and Medawar to contemporary clinical experience with CTSA. In all cases, the progressive disappearance of the allogeneic epidermis was accompanied by the relative persistence of the dermis and transient preservation of the extracellular matrix architecture. In more recent clinical models, including CTSA, these phenomena are associated with vascularization, cellular infiltration, and tissue remodeling, suggesting that immunological and reparative processes may coexist during graft evolution.

These observations raise fundamental questions. If a structurally preserved extracellular matrix and an active but regulated immune response can coexist during the evolution of skin allotransplantation, rejection may not be an exclusively destructive mechanism. Under certain conditions, it may also constitute a transitional phase capable of generating the biological conditions required to activate latent regenerative programs in the skin.

The hypothesis proposed in this study emerges precisely from this paradox. Rather than interpreting rejection as the inevitable failure of the graft, we propose that the interaction between a preserved extracellular matrix and a controlled immune response can generate a microenvironment capable of directing tissue regeneration.

## 6 Immunoguided cutaneous regeneration: a theoretical framework

**Figure 1:**
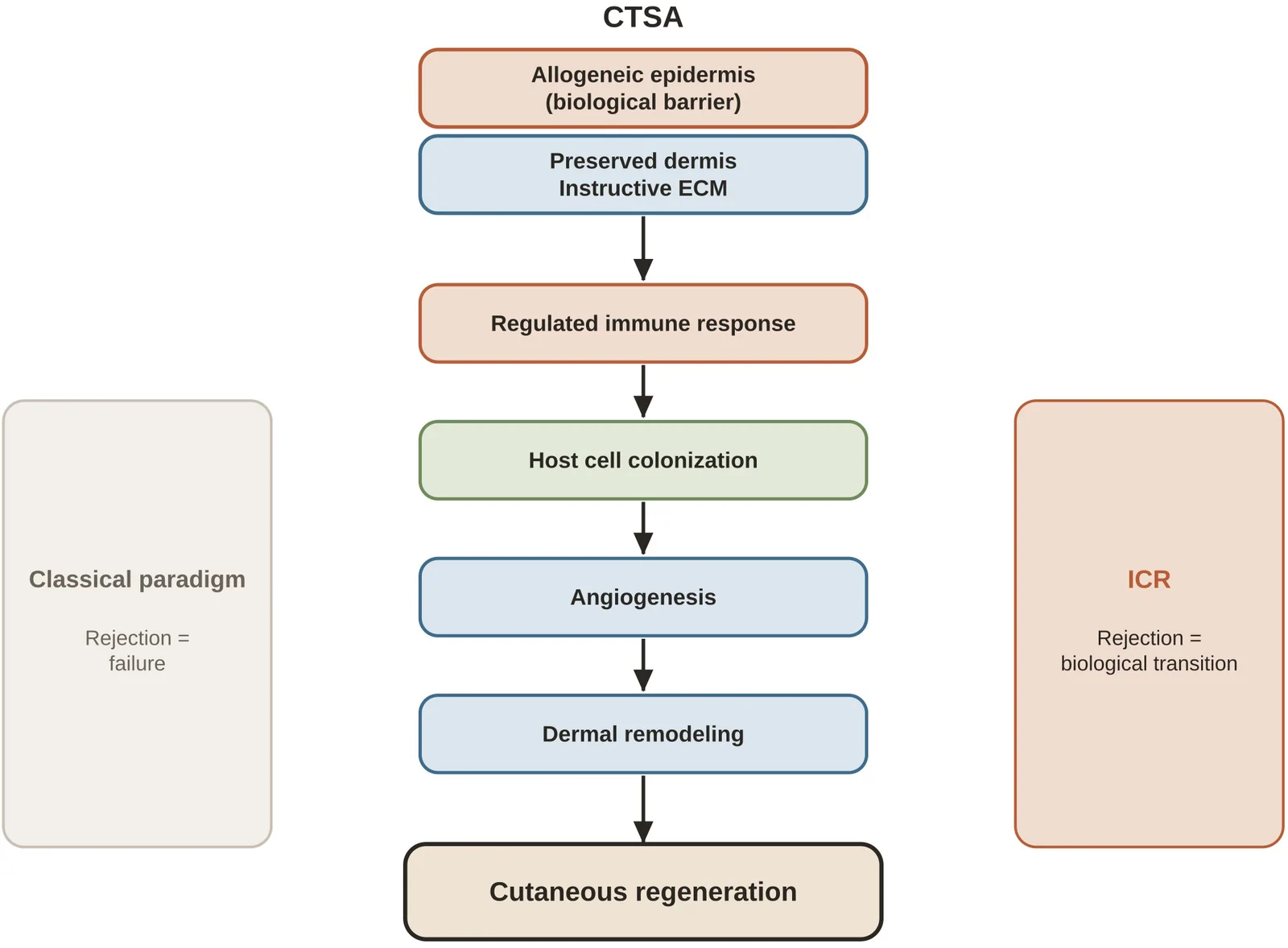
Conceptual model of immunoguided cutaneous regeneration. The bicompartmental allograft, comprising an allogeneic epidermis as a biological barrier and preserved dermis as an instructive extracellular matrix, triggers a regulated immune response that orchestrates host cell colonization, angiogenesis, and dermal remodeling, culminating in skin regeneration. Unlike the classical paradigm (rejection = failure), the ICR reinterprets rejection as a biological transition.

ICR is a theoretical framework proposing that tissue regeneration is an emergent property of a transient immunoregenerative microenvironment generated by the interaction between a preserved extracellular matrix and a regulated host alloimmune response (**Figure 1**). Within this framework, regeneration does not depend primarily on the persistence of viable donor cells but on the ability of the graft to transiently recreate the biological conditions that allow the latent regenerative programs of the host to be expressed.

Therefore, the central postulate of ICR is that regeneration emerges from the coordinated interaction between the preserved extracellular matrix and regulated alloimmunity rather than from either component alone. The preserved allogeneic matrix provides structural, biomechanical, and biochemical cues, whereas the host immune response coordinates cellular recruitment, angiogenesis, matrix remodeling, and ultimately, the resolution of repair. Regeneration is thus interpreted as a systems-level property arising from the interaction between the graft-derived matrix and the recipient, rather than as a direct consequence of residual biological activity within the graft itself.

**Figure 2:**
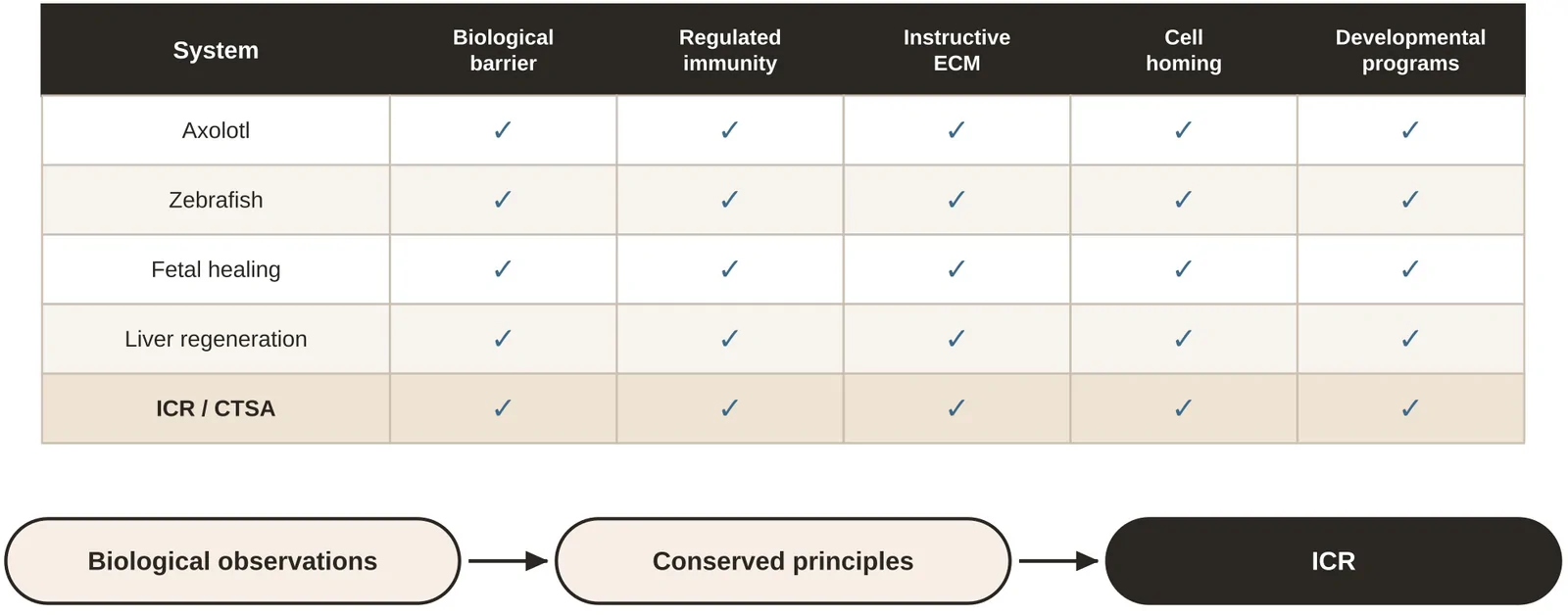
Conserved regenerative principles and conceptual origins of ICR. Five biological principles—protective barriers, regulated immunity, instructive extracellular matrix, guided cell recruitment, and reactivation of developmental programs—converge across highly regenerative organisms (axolotl, zebrafish), human regenerative phenomena (fetal healing, liver regeneration), and repair mediated by cryopreserved total skin allografts (CTSA). The ICR does not arise from the CTSA but from the generalization of conserved principles.

The ICR framework is based on the observation that regenerative systems described in phylogenetically distant organisms share a relatively conserved biological organization. Five fundamental principles consistently emerge: (i) establishment of a temporary biological barrier; (ii) activation of a transient and tightly regulated innate immune response; (iii) preservation of an extracellular matrix capable of providing structural, biomechanical, and biochemical cues; (iv) coordinated recruitment (homing) of reparative cells from neighboring tissues or circulation; and (v) transient reactivation of molecular programs normally associated with embryonic development. Rather than representing isolated events, these principles interact to generate a microenvironment permissive for regeneration (9, 10, 13, 16) (**Figure 2**).

We propose that, in the context of CTSA-mediated repair, these conserved regenerative principles are temporally organized into four partially overlapping phases that together constitute the cutaneous expression of this biological program. Initially, the allogeneic epidermis provides a temporary biological barrier, whereas the preserved dermis maintains an organized extracellular matrix that supports host cell infiltration and tissue organization. Rather than opposing tissue repair, the alloimmune response coordinates cellular infiltration, angiogenesis, matrix remodeling, and subsequent resolution. Under ICR, immunity is interpreted not as an obstacle to regeneration but as one of its principal biological organizers.

Within this framework, regeneration is proposed to occur not despite the alloimmune response but through a temporally regulated alloimmune response that is initiated, coordinated, and ultimately resolved. Therefore, the biological distinction lies not in whether inflammation occurs but in whether it progresses toward physiological resolution or persists as self-sustaining immune–fibrotic activation. The immunological substrate supporting this hypothesis is developed below, followed by the four regenerative phases through which the proposed mechanism operates.

## 7 The immunological substrate of immunoguided cutaneous regeneration

ICR assigns immunity a causal rather than an incidental role, and a theory that makes this claim must specify which immunological events it invokes, in what order, and under what conditions they would fail to occur. We use the term regulated alloimmune response in an operational sense: a response of sufficient magnitude to clear the antigenic burden of the graft, yet of limited duration and cytotoxic amplitude, followed by effective resolution, such that both immunological silence and unrestrained alloreactivity would be incompatible with regeneration.

### 7.1 Innate initiation

Cryopreservation and terminal irradiation at 25 kGy deliver structurally intact but biologically non-viable tissue, whose implantation constitutes a sterile inflammatory stimulus: constituents released by devitalized cells act as damage-associated molecular patterns recognized by host receptors, initiating neutrophil and monocyte recruitment without a microbial trigger (45). We propose that this early sterile inflammatory phase is not incidental but the first organizing event of ICR, initiating the immune cascade that subsequently coordinates debris clearance, matrix remodeling, angiogenesis, and tissue regeneration.

### 7.2 Macrophages and the matrix interface

Macrophages occupy a pivotal position in this transition, contributing to debris clearance, angiogenesis, matrix remodeling, and recruitment of reparative populations (17, 18). We retain the M1/M2 terminology as a descriptive shorthand for the two poles of a continuum rather than as discrete categories, since the current consensus recognizes macrophage activation as a spectrum shaped by tissue origin, stimulus, and temporal context (46). ICR proposes something more specific: that the preserved dermal matrix participates in determining the location of host macrophages along this continuum. Matrix degradation releases matricryptic peptides and sequestered factors, and biological scaffolds carry matrix-bound nanovesicles capable of directly modulating the myeloid phenotype (47). The observation that a pro-regenerative response to such scaffolds requires type 2 immunity (25) suggests that this interface is not merely permissive but also instructive.

### 7.3 Allorecognition in a devitalized allograft

The adaptive component of ICR fundamentally differs from that of conventional allotransplantation. Direct allorecognition depends on viable passenger leukocytes, the depletion of which markedly reduces graft immunogenicity (48). Cryopreservation and irradiation eliminate donor cell viability, including that of dendritic cells and functional donor endothelium. Because donor alloantigens persist within non-viable tissue remnants, we propose that allorecognition in CTSA occurs predominantly through the indirect pathway, restricted to MHC class II presentation and mediated primarily by CD4+ T cells (49), with the semi-direct pathway also attenuated (50). Therefore, the resulting immune response should differ from classical acute rejection: slower, lower in cytotoxic amplitude, and directed toward progressive clearance of antigenic material rather than rapid tissue destruction, consistent with the histological evolution observed in CTSA. Under these conditions, the effector phase does not rely predominantly on CD8+ cytotoxicity. CD4+-dependent, macrophage-mediated mechanisms may contribute substantially to graft clearance, and phagocytic removal of devitalized tissue is more compatible with the preservation of collagen architecture than destruction mediated primarily by cytotoxic granules.

### 7.4 Resolution and the tolerance–fibrosis tension

Regeneration requires not only the occurrence of the alloimmune response but also its resolution. Regulatory T cells, together with IL-10 and TGF-β, are central mediators of alloresponse resolution (51), and polarization toward regulatory immune programs has been associated with reduced alloreactivity and improved graft integration (52). This phase introduces a defining biological tension: TGF-β is simultaneously a principal mediator of immune tolerance and one of the strongest drivers of fibrosis (19). Therefore, ICR proposes that the preserved dermal matrix provides a local counterbalance through retained decorin and fibromodulin, which bind and modulate TGF-β signaling (53). Consequently, the regenerative window is predicted to be narrow, transient, and critically dependent on the balance between immune resolution and fibrosis activation.

### 7.5 Delimitation: chronic rejection

Chronic rejection, classically defined by allograft vasculopathy, requires persistent donor endothelium within a surgically vascularized graft (54, 55). None of these conditions apply to CTSA, where donor endothelial cells are non-viable and the original microvasculature is progressively replaced by recipient vessels. Therefore, we considered chronic rejection to be outside the scope of the present model. Instead, the biologically relevant failure mode for CTSA is predicted to be excessive fibrosis, an outcome specifically addressed by the predictions developed in Section 10.

The regenerative phenomena motivating ICR also admit alternative explanations, including the effects of biological dressings, passive release of matrix-bound growth factors, and constructive remodeling of extracellular matrix (ECM) scaffolds. The ICR does not exclude these mechanisms; rather, it proposes that they are insufficient to explain regeneration in the absence of a temporally regulated alloimmune response. Its distinguishing feature is the hypothesis that regulated alloimmunity is causally required, rather than merely permissive, for successful regeneration in the adult.

## 8 Phases of immunoguided cutaneous regeneration

The ICR framework proposes that the regenerative response observed following the application of CTSA is not a single biological event but a temporal expression of a transient immunoregenerative microenvironment generated through the interaction between a preserved extracellular matrix and a regulated host alloimmune response. This process represents the temporal evolution of the microenvironment, through which a devitalized allogeneic scaffold is transformed into recipient-integrated regenerative tissue.

**Figure 3:**
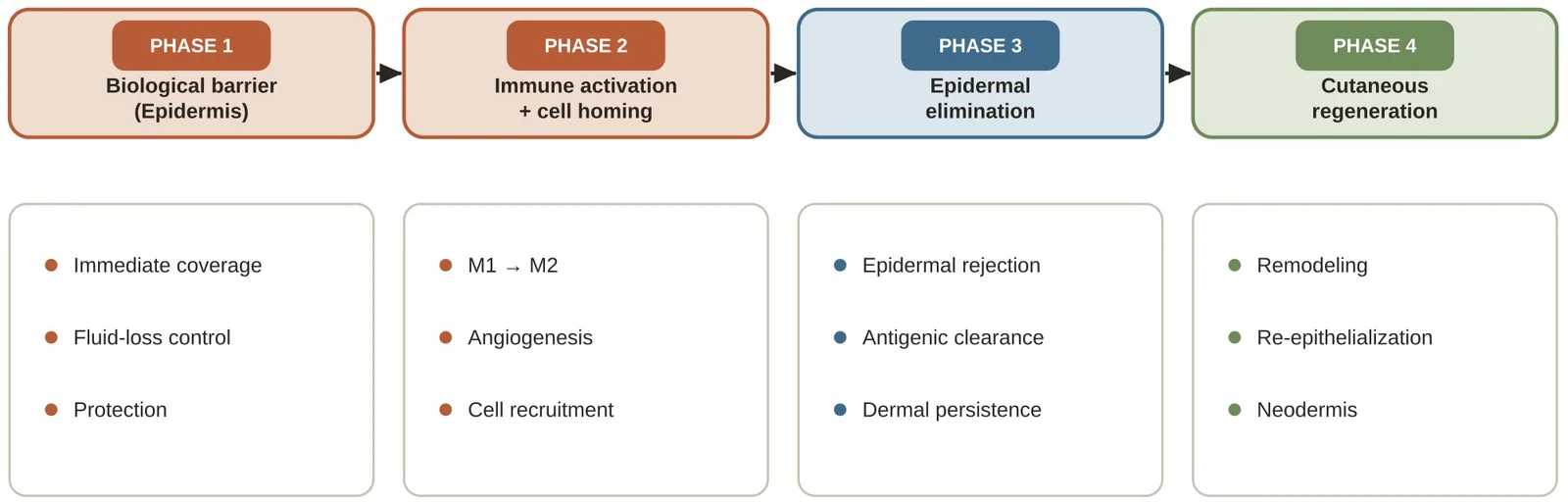
The four phases of immunoguided cutaneous regeneration. Phase 1: Biological barrier provided by the epidermis (immediate coverage, fluid loss control, protection); Phase 2: Immune activation and cell recruitment (M1→M2 polarization, angiogenesis); Phase 3: Progressive elimination of the epidermal component (epidermal rejection, antigenic clearance, dermal persistence); Phase 4: Cutaneous regeneration (remodeling, re-epithelialization, neodermis). The phases partially overlapped.

Based on the historical, experimental, and clinical evidence reviewed in this article, we propose four functional phases: (i) establishment of a biological barrier, (ii) immune activation and cellular colonization, (iii) selective elimination of the epidermal component, and (iv) regenerative remodeling (**Figure 3**). Each phase is defined by its predominant biological function; however, none occurs in isolation, and together, they establish, maintain, and ultimately resolve the immunoregenerative microenvironment. For descriptive purposes, they are presented sequentially; however, the transitions are gradual, reflecting the evolving balance between matrix preservation, regulated immunity, cellular recruitment, angiogenesis, and remodeling that determines whether repair advances toward constructive regeneration or fibrosis. Moreover, each phase yields experimentally testable consequences, developed as the predictions of Section 10.

### 8.1 Biological barrier phase

The first phase involves the rapid establishment of a temporary biological barrier that creates the conditions required for the subsequent immunoregenerative microenvironment. Rather than serving solely as a physical covering, this barrier limits fluid loss, reduces microbial contamination, buffers exposure to external stimuli, and stabilizes the local wound environment. Across multiple regenerative systems, including urodele amphibians and zebrafish, the early formation of epithelial covering is an indispensable prerequisite for the activation of regenerative programs (8, 16).

In the classical model of urodele limb regeneration, the wound epidermis gives rise to the apical epithelial cap (AEC), a specialized transient structure that protects the underlying tissue and regulates blastema formation, cellular proliferation, and the molecular signaling required for regeneration. Experimental removal of the AEC prevents normal limb regeneration, demonstrating that epithelial covering functions well beyond a simple physical barrier (8).

Although the CTSA does not literally reproduce this system, we propose that it preserves one of its fundamental principles: the early establishment of a temporary interface that protects the wound while creating a permissive environment for the events that follow. Immediately after implantation, the epidermal surface provides biological coverage, while the intact dermal architecture stabilizes the wound, isolating it, reducing fluid loss, and creating the local conditions required for the development of an immunoregenerative microenvironment. These events precede angiogenesis, colonization, immune regulation, and matrix remodeling, which characterize the later phases.

Within ICR, the biological significance of the epidermal component lies not in its persistence but in its capacity to initiate the sequence through which the microenvironment is established. Similar to other transient structures observed during regeneration, its role is organizational rather than permanent; as it disappears, the dermal scaffold assumes the dominant role, providing the framework within which the regulated alloimmune response coordinates remodeling and drives regeneration.

### 8.2 Immune activation and cellular colonization phase

Once the barrier has stabilized the wound, the regenerative process becomes critically dependent on the interaction between the preserved dermal matrix and host immune response. In ICR, immune activation is not an obstacle to regeneration but rather the mechanism through which the immunoregenerative microenvironment is established. Rather than solely eliminating damaged tissue or foreign material, the host response orchestrates the transition from a devitalized allogeneic graft toward an environment capable of supporting constructive remodeling.

This microenvironment is closely linked to innate immune activation across virtually all regenerative systems. Following injury, inflammatory cells coordinate debris clearance, initiate matrix remodeling, promote angiogenesis, recruit reparative cells, and regulate the signals that determine whether repair advances toward regeneration or fibrosis. Successful regeneration therefore depends not on the absence of inflammation but on its precise temporal regulation: the biological determinant is not whether inflammation occurs, but whether it resolves physiologically or evolves into self-sustaining immune–fibrotic activation (9, 10, 17, 18).

This principle is supported by regenerative biology. Pathological wound healing is now recognized not as a consequence of fibroblast-autonomous dysfunction but as a result of persistent immune–stromal interactions that sustain chronic inflammatory and profibrotic signaling. In hypertrophic scars and keloids, sustained immune activation, continuous cytokine production, and prolonged fibroblast stimulation prevent the normal transition toward remodeling, resulting in excessive matrix accumulation (56, 57). The defining feature of these conditions is not the presence of inflammation, which is indispensable for all repairs, but the failure of its resolution (56, 58). Notably, this chronic activity localizes predominantly within the reticular dermis, whereas superficial injuries that spare this compartment rarely scar pathologically (59). By restoring the complete dermal architecture, CTSA precisely reconstitutes the compartments implicated in these conditions.

These observations define the principal boundary condition of the theory, rather than contradicting it. ICR does not propose that a preserved matrix is intrinsically regenerative; rather, it proposes that regeneration becomes possible only when the preserved dermal architecture and a temporally regulated immune response interact to establish a constructive microenvironment. An identical matrix implanted in an environment of persistent immune activation is predicted to yield fibrosis rather than regeneration. This contrast is empirically verifiable and is stated as a direct test in Prediction 3.

Within this network, macrophages act as the central coordinators of remodeling. Beyond phagocytosis and antimicrobial defense, they regulate angiogenesis, matrix remodeling, recruitment of reparative populations, and the transition from inflammation to reconstruction. Experimental depletion of these factors impairs regeneration across multiple systems, including amphibian limbs (9), zebrafish hearts (60), livers (22), and skin (10). This interaction is reciprocal; as inflammatory cells remodel the dermal scaffold, matrix degradation releases matricryptic peptides and sequestered factors that shape macrophage activation and, in turn, the local immune environment (12, 20). Neither immunity nor the matrix acts independently; each modifies the behavior of the other, generating reciprocity that establishes an immunoregenerative microenvironment.

Studies on biological scaffolds reinforce this view. Badylak, Brown, and others showed that preserved matrices steer the host response toward phenotypes associated with constructive remodeling, accompanied by angiogenesis, organized remodeling, resolution of inflammation, and reduced fibrosis. Comparable observations in skin allograft models associate polarization toward regulatory programs with reduced alloreactivity and improved integration (17, 18, 52). Thus, preserved matrices not only provide structural support but also shape the biological quality of repair (34, 35).

The CTSA provides a distinctive context in which these mechanisms unfold. Cryopreservation followed by terminal irradiation at approximately 25 kGy eliminates donor cellular viability and markedly reduces immunogenicity while preserving the three-dimensional organization of the dermal matrix. The biological activity observed after implantation is therefore interpreted not as the persistence of viable donor cells but as the recipient’s response to a structurally preserved matrix that retains its architectural and biochemical cues. Experimental studies support this: allografts irradiated at this dose retain tissue organization and continue to support angiogenesis, cellular infiltration, granulation, and collagen deposition (61, 62). Notably, Tomaz de Miranda et al. found that grafts irradiated at 25 kGy showed reduced dermoepidermal necrosis, diminished neutrophilic infiltration, enhanced neovascularization, and superior repair relative to higher doses (61), consistent with an environment in which preserved architecture and regulated immunity together promote constructive remodeling.

Early matrix remodeling and restored vascularization then enable the coordinated recruitment (homing) of host cells, allowing inflammatory cells, endothelial cells, fibroblasts, and progenitors to migrate along chemotactic gradients and matrix-derived signals (13). The graft thereby ceases to behave as an isolated allogeneic tissue and integrates into the recipient’s biological network. Colonization follows as recipient cells repopulate the dermal scaffold, residual donor remnants are cleared by the regulated alloimmune response, and the two processes proceed concurrently, shifting the graft’s identity from devitalized allogeneic tissue toward a recipient-integrated regenerative template. This transition does not replace the scaffold but repopulates it; the matrix continues to guide migration, vascular ingrowth, and remodeling, while immunity regulates the magnitude and tempo of these events. Immune activation and colonization are thus inseparable aspects of a single process that establishes the conditions for selective epidermal elimination.

### 8.3 Selective epidermal elimination phase

Within ICR, the disappearance of the epidermal component represents neither graft failure nor complete rejection but a predictable transition reflecting the differential fate of the two compartments of CTSA. Following implantation, the epidermal component undergoes necrosis and is eliminated as donor antigenicity resolves, whereas the dermal architecture remains integrated within the wound. Rather than reflecting complete graft rejection, this represents differential remodeling of the two compartments and is one of the defining features of ICR (9, 10, 17, 18).

This differential behavior is consistent with the distinct immunological composition of the compartments. The epidermis contains the highest density of donor-derived antigen-presenting cells, particularly Langerhans cells, and keratinocytes act as non-professional antigen-presenting cells under inflammatory conditions. Therefore, the epidermis is the principal source of residual donor antigenicity, participating predominantly in host allorecognition through direct, indirect, and semi-direct pathways (49, 50). The dermis is not immunologically inert; it contains the vascular endothelium, dermal dendritic cells, resident lymphocytes, and other stromal elements. However, terminal irradiation renders these cells non-viable while preserving the three-dimensional matrix, reducing immunogenic potential without disrupting structural organization (17, 18, 34, 35, 61, 62).

The experimental evidence supports this interpretation. Wu et al. demonstrated that preservation of native matrix architecture may physically shield residual immunogenic cells while maintaining the structural integrity required for constructive remodeling (63). Therefore, this organization may influence not only the mechanical behavior of the graft but also the spatial presentation of residual alloantigens to the host, offering a mechanistic explanation for the preferential loss of the epidermal component alongside dermal preservation. Within the ICR, this is interpreted as one of the structural factors sustaining the immunoregenerative microenvironment rather than an isolated immunological phenomenon.

Clinical observations are consistent with this hypothesis. Following implantation, the epidermal layer becomes necrotic, forms a superficial eschar, and detaches, while the dermal component remains firmly adherent to the wound bed (27, 28, 64); during this period, vascular ingrowth, recipient-cell infiltration, matrix remodeling, and structural integration occur together within the preserved dermis (13, 61, 62). Selective epidermal elimination should therefore be read as a functional transition rather than an endpoint of rejection: the epidermal compartment fulfills a temporary organizational role, and its disappearance coincides with the resolution of residual antigenicity and the maturation of the reparative environment, while the dermal matrix continues to supply the cues required for angiogenesis, host-cell migration, remodeling, and organization (17, 18, 34, 35).

This resembles developmental processes in which transient structures are removed after fulfilling their organizational functions, while the underlying program continues uninterrupted (8, 16). The persistence of regenerative activity despite epidermal loss supports the view that regeneration is sustained by preserved dermal architecture and the immunoregenerative microenvironment established earlier. Selective epidermal elimination thus forms a bridge between immune-mediated graft adaptation and definitive reconstruction, leading to the final regenerative phase.

### 8.4 Regenerative remodeling phase

The final phase is characterized by the transformation of the integrated dermal scaffold into a functional neodermis through matrix remodeling, cellular differentiation, vascular maturation, and tissue organization processes. By this stage, the temporary barrier has disappeared, the immunoregenerative microenvironment is established, recipient-derived cells have substantially repopulated the scaffold, and the residual donor antigenic burden has largely resolved. Tissue reconstruction therefore no longer depends on viable donor cellular elements but on the capacity of the integrated matrix to direct host-driven regeneration (13, 17, 18, 34, 35).

Here, the dermal matrix functions as a biologically instructive scaffold rather than as an inert support. Its three-dimensional organization provides structural, biomechanical, and biochemical cues that regulate cell migration, vascular maturation, fibroblast organization, and collagen remodeling. Rather than instructing regeneration independently, the matrix influences the behavior of recipient cells, which in turn remodel their surrounding environment, an instance of the dynamic reciprocity originally proposed by Bissell, whereby matrix architecture regulates cell behavior while cells remodel the matrix (11, 12, 20, 32, 33). Within the ICR, this reciprocity extends beyond classical cell–matrix communication by incorporating the sustained influence of the immunoregenerative microenvironment. As immune activity resolves and homeostasis is restored, remodeling, vascular maturation, and differentiation become coordinated, enabling the reconstruction of the normal dermal architecture. The divergence between regeneration and fibrosis may depend on the composition of the preserved dermis, which retains small leucine-rich proteoglycans, such as decorin and fibromodulin, that bind and modulate TGF-β signaling, thereby counterbalancing myofibroblast activation and disorganized collagen deposition, which are characteristic of scarring (53). Preservation of full dermal thickness may be biologically relevant, as CTSA retains both papillary and reticular dermis with compositional gradients of these regulators. The instructive element is not represented by donor fibroblasts, which are ultimately removed for their cellular and antigenic nature, but by the architectural and biochemical information embedded within the matrix.

The consequence is not the persistence of the implanted allograft but its gradual transformation into recipient-integrated tissue: the original scaffold loses its identity as an allogeneic matrix as it is incorporated into a host-generated extracellular network, functioning as a temporary regenerative template until its properties merge with the newly formed dermis. This is consistent with the clinical behavior of the CTSA. After separation of the epidermal eschar, the underlying tissue shows vascularization, increased mechanical stability, restored dermal thickness, and the capacity to support definitive epithelialization through spontaneous epithelial migration or subsequent split-thickness grafting. Histologically, the stage shows organized collagen deposition, matrix remodeling, vascular maturation, and dermal organization consistent with constructive remodeling rather than fibrotic scarring (61, 62).

Within the ICR, regeneration is an emergent systems-level property arising from the coordinated interaction among preserved matrix architecture, regulated immunity, vascular restoration, cellular colonization, and continuous remodeling. No single component suffices; functional reconstruction emerges from their integration over time, and the formation of a functional neodermis is the final expression. Across the four phases, a devitalized allograft is converted into a recipient-integrated template capable of supporting a functional neodermis, with overlapping stages in the establishment, maintenance, and resolution of the immunoregenerative microenvironment. Crucially, this sequence is not merely descriptive; it generates experimentally testable predictions. Among them, the proposition that a regulated alloimmune response is causally required, rather than merely permissive, most clearly distinguishes the ICR framework from purely structural models of tissue repair.

## 9 ICR as a unifying framework for previously unconnected observations

One of the defining characteristics of a robust biological theory is not simply its capacity to propose new mechanisms but its ability to organize previously independent observations into a coherent explanatory framework. Rather than replacing existing evidence, successful theories reveal underlying relationships that are not apparent when individual findings are considered in isolation. The studies discussed below span more than eight decades of research in skin allotransplantation, extracellular matrix biology, regenerative medicine, and reconstructive surgery; they were performed in different species using distinct experimental models and were never intended to test a common hypothesis. Collectively, however, they reveal a recurring biological pattern that has remained conceptually fragmented (**Table 1**).

**Table 1:** Evidence supporting the immunoguided cutaneous regeneration (ICR) theory.

Observation	Author	Main finding	ICR interpretation
Dermal persistence	Gibson and Medawar	The dermis persists longer than the epidermis	Differential fate
Matrix immunoprotection	Wu et al.	Dermal architecture limits infiltration	Immunological accessibility
Sandwich phenomenon	Yang et al.	Autologous epidermis over allogeneic dermis	Coexisting rejection and repair
Functional dermis	Cuono et al.	Dermo-epidermal reconstruction	Competent biological scaffold
Constructive remodeling	Badylak et al.	M2 and functional repair	Pro-regenerative immunity
CTSA	Fonseca et al.	Neodermis and clinical regeneration	Clinical application of the model

Six historical and clinical observations (Gibson and Medawar, Wu, Yang, Cuono, Badylak, and Fonseca), with their main finding and their reinterpretation under the ICR model.

From the perspective of ICR, these apparently unconnected observations become components of a single regenerative sequence, describing complementary stages of a common process in which a preserved extracellular matrix interacts with a regulated host immune response to generate a transient immunoregenerative microenvironment that ultimately supports tissue regeneration.

Taken together, these observations exhibit a degree of biological convergence, or consilience, that would not be expected if they reflected entirely independent phenomena; the strength of ICR lies not in explaining any single observation uniquely, but in offering a coherent framework capable of integrating them while generating experimentally falsifiable predictions.

### 9.1 Gibson, Medawar, and Wu: early evidence of differential compartmental biology

The earliest observations supporting the ICR framework emerged decades before the concepts of regenerative immunology and extracellular matrix biology were established. Within the classical paradigm of skin allotransplantation, the differential behavior of the epidermis and dermis during graft rejection was regarded primarily as a descriptive feature of graft evolution, rather than as evidence that the two compartments fulfilled distinct biological functions. The ICR offers a different interpretation, viewing these early observations as the first indications that the epidermis and dermis follow divergent biological trajectories during tissue repair.

In their pioneering studies on skin transplantation, Gibson and Medawar observed that the epidermis underwent rapid rejection, whereas the dermal component persisted considerably longer, maintaining vascularization and much of its structural organization despite the ongoing immune response. They further described that dermal collagen bundles were remarkably resistant to destruction and were removed gradually through erosion and phagocytosis (41). At the time, this differential persistence was largely interpreted as a characteristic of rejection kinetics. The possibility that the retained dermal matrix might actively influence subsequent tissue repair was not considered, reflecting the prevailing view of the extracellular matrix as a passive structural framework rather than a biologically instructive component of the skin.

Several decades later, Wu et al. provided a potential mechanistic explanation for this. Their work demonstrated that the native three-dimensional organization of the dermal extracellular matrix can physically limit lymphocyte access to resident immunogenic cells while preserving tissue structural integrity (63). These findings suggest that the matrix architecture influences not only the mechanical properties of the graft but also the spatial organization of immune recognition. Consequently, the extracellular matrix emerges as an active regulator of the biological context in which the host immune response develops, rather than merely being the residual scaffold remaining after cellular rejection.

Taken together, Gibson and Medawar documented the phenomenon, and Wu proposed a mechanism to explain it: the preserved dermal matrix is not simply the residual component of a rejected graft but an active determinant of its subsequent biological fate. Therefore, the preferential persistence of the dermal compartment is an early manifestation of differential compartmental biology that gives rise to the immunoregenerative microenvironment proposed in this theory.

### 9.2 Yang and Cuono: coexistence of rejection and dermal competence

Two historical observations converge on the same conclusion: the allogeneic dermis remains biologically functional during rejection. Yang et al. described the sandwich phenomenon, in which autologous epidermis progressively replaces the rejected allogeneic epidermis while the allogeneic dermis remains integrated within the wound bed (42). The conventional interpretation regards this as a strategy to expand autologous coverage. From the perspective of ICR, it acquires an additional dimension: selective epidermal elimination and preservation of a functional dermal scaffold are not mutually exclusive processes but are complementary components of the same regenerative sequence. Thus, the sandwich phenomenon anticipates, in a clinical setting, the differential compartmental biology developed in Section 8. Cuono et al. took this observation one step further. By deliberately removing the alloepidermis to expose the engrafted allogeneic dermis, they reproduced a sequence remarkably similar to the biological transition proposed by ICR to occur spontaneously in a controlled surgical setting. They applied cultured autologous keratinocytes onto this vascularized dermal scaffold, successfully reconstructing a functional dermoepidermal junction (43). The experiment demonstrated that the retained dermis was not a mere structural remnant but a biologically competent substrate capable of supporting definitive tissue reconstruction. Although Cuono achieved epidermal restoration through cultured keratinocytes rather than spontaneous epithelial migration, both situations share the same biological prerequisite that ICR posits: the persistence of a competent dermal scaffold after the disappearance of the epidermal component of the wound.

### 9.3 Acellular dermal matrices: extending an established biological principle

The regenerative capacity of the preserved dermal extracellular matrix is well established in reconstructive surgery and does not originate from the ICR framework. Human acellular dermal matrices (ADMs), such as AlloDerm, have consistently demonstrated that preserved extracellular matrix can support host cell infiltration, angiogenesis, vascular maturation, and long-term remodeling into a functional neodermis (65). These products established an important principle of regenerative medicine: biological matrices can actively guide tissue reconstruction, even in the absence of viable donor cells. Rather than contradicting this principle, the ICR extends it to a different biological context. Both ADMs and CTSA ultimately function as recipient-integrated regenerative scaffolds, whose biological activity derives primarily from preserved dermal architecture rather than from long-term donor cell viability. The distinction proposed by the ICR is therefore not the regenerative role of the extracellular matrix itself but the biological pathway through which this scaffold is established. In ADMs, donor cellular components are removed before implantation through ex vivo decellularization, a process designed to minimize alloimmune activation while preserving sufficient matrix architecture to permit host colonization of the graft. The scaffold is thus implanted in an essentially acellular state, and regeneration proceeds largely through host repopulation without substantial immune recognition of the donor antigens. CTSA follows a fundamentally different trajectory: terminal irradiation devitalizes donor cells while preserving the native, full-thickness organization of the skin. Although cellular remnants initially remain within the tissue, they progressively disappear through immune-mediated physiological clearance after transplantation. Within the ICR, this process is interpreted not as a mere consequence of implantation but as an integral component of the regenerative sequence itself: rather than preventing alloimmune activation, CTSA permits a controlled, self-limited immune response that removes devitalized donor remnants while preserving the scaffold that supports regeneration. The biological status of CTSA warrants precision: although the term “viable” was used in its initial clinical characterization (64), this interpretation was subsequently refined, with CTSA recognized as corresponding better to a structurally preserved, biologically active matrix rather than a cellularly viable tissue (66).

This distinction has important biological implications. In ADMs, regeneration begins after immune minimization is achieved through prior decellularization. In CTSA, immune activation and tissue regeneration occur simultaneously within the preserved native scaffolds. ICR proposes that this transient interaction between the host immune system and the preserved dermal architecture contributes to establishing an immunoregenerative microenvironment, such that immune-mediated cellular clearance is not merely a permissive event but a process that may actively coordinate vascularization, matrix remodeling, and recipient cell integration. Whether this contribution is causally required rather than merely associated with regeneration constitutes one of the experimentally testable predictions generated by the model.

The second distinction concerns the preservation of tissue architecture. As ex vivo decellularization necessarily employs detergents, enzymatic digestion, or other extraction procedures, some degree of matrix modification is unavoidable despite careful optimization. In contrast, terminal irradiation devitalizes cellular elements while largely preserving the native three-dimensional organization of the dermis, including the collagen architecture, basement membrane relationships, and full-thickness structural gradients. Although irradiation also induces measurable biochemical alterations in collagen cross-linking, as discussed in Section 11.7, the overall preservation of tissue architecture may provide a more faithful structural template for recipient cell migration and remodeling.

The ICR does not propose that CTSA replaces or surpasses acellular dermal matrices, but that both represent complementary biological solutions based on the same fundamental principle: regeneration is orchestrated by preserved extracellular matrix rather than by sustained donor-cell survival. The difference lies in how this scaffold is established: ADMs reach this state before implantation through ex vivo decellularization and immune minimization, whereas CTSA reaches a comparable endpoint through *in vivo* immune-guided remodeling after implantation. Under this interpretation, CTSA does not constitute a concept opposed to acellular dermal matrices but a biological extension of the same principle: an instructive scaffold whose acellular condition is attained through immune guidance rather than immune evasion and whose architectural integrity is preserved through devitalization rather than extraction.

### 9.4 Badylak and the transition from the immunology of rejection to the immunology of regeneration

Investigations by Badylak and other authors introduced a profound conceptual shift by demonstrating that the immune response determines not only the destruction of implanted tissues but also the quality of the resulting repair process. The observation that certain extracellular matrices can induce immune responses associated with constructive remodeling, angiogenesis, and functional repair provides one of the most important conceptual pillars for ICR (34, 35). In this context, immunity ceases to be interpreted exclusively as a destructive force and is understood as an active regulator of the biological fate of the injured tissues. The theory proposed in this study extends this principle to skin allotransplantation, suggesting that the interaction between a preserved dermal matrix and a regulated alloimmune response may generate an immunoregenerative microenvironment capable of favoring regenerative processes, even in the presence of an active immune response.

### 9.5 Tomaz de Miranda: experimental evidence from irradiated human skin

Among the available experimental observations, the studies by Tomaz de Miranda are particularly relevant because of their biological proximity to this proposed model. Using human skin allografts irradiated at 25 kGy, the authors observed cellular infiltration, neovascularization, and a biological profile compatible with constructive remodeling (61).

These findings warrant important qualifications. The model employed, a human skin xenograft implanted into athymic mice lacking functional T lymphocytes, cannot, by its own design, test the alloimmune component that ICR posits as causal; what it demonstrates is that the irradiated human dermal matrix retains its instructive capacity and permits constructive remodeling even in the absence of a T-cell response. Consequently, from the perspective of ICR, this study specifically supports the matrix-instructive component of the model, which states that irradiation does not eliminate the biological capacity of the matrix to interact with host cells, whereas the immune component must be evaluated in immunocompetent systems, as posed in Prediction 7.

### 9.6 CTSA as an integration of the elements of the model

The clinical observations obtained with the CTSA appear to integrate many of the phenomena previously described independently within a single scenario. Initial graft engraftment, appearance of a superficial epidermal eschar, persistence of a vascularized dermis, cellular infiltration, tissue remodeling, and formation of a neodermis sequentially reproduce the principal elements proposed by this theory.

From this perspective, the CTSA does not merely constitute a new modality of biological coverage. It represents a clinical model capable of bringing together within a single process phenomena that had historically been observed separately: the dermal persistence described by Gibson and Medawar (41), the modulation of immunological accessibility proposed by Wu (63), the coexistence of epidermal elimination and dermal function observed by Yang (42), the reconstructive capacity demonstrated by Cuono (43), and the constructive remodeling associated with biological matrices described by Badylak (34, 35). The preservation of the full-thickness dermis, developed in Section 8.4, may explain why these regenerative phenomena, appearing only partially or indirectly in historical studies using split-thickness grafts, have become more evident in contemporary CTSA applications. Rather than being a distinct biological phenomenon, CTSA represents a more complete expression of the same underlying process.

The initial clinical series with CTSA further illustrated two biological trajectories compatible with the regenerative phase proposed by the ICR. In some patients, the neodermis generated following the immunological transition enabled definitive reconstruction through secondary autografting, constituting an assisted regeneration model. In others, progressive remodeling culminated in the spontaneous restoration of cutaneous continuity without additional coverage, constituting a model of complete regeneration (27, 28). Although these observations do not demonstrate the proposed mechanism, they are consistent with the two regenerative trajectories predicted by this theory. The clinical pattern observed with CTSA is consistent with the sequence proposed by the ICR and was one of the principal stimuli for its formulation; however, it should be understood as compatible with the model rather than as a demonstration of it. A clinical series may illustrate a biological mechanism, but it cannot definitively establish it.

The evidence reviewed in this section converges toward a common biological interpretation; however, this convergence constitutes consilience rather than proof. These are heterogeneous observations obtained from different experimental models and clinical contexts, each with its own methodological limitations. The principal value of ICR lies not in the fact that this evidence conclusively demonstrates it, but in its capacity to provide a conceptual framework capable of integrating such observations into a coherent explanation and, above all, generating specific and potentially falsifiable predictions regarding the interaction between immunity, the extracellular matrix, and cutaneous regeneration.

## 10 Testable predictions of immunoguided cutaneous regeneration

A defining characteristic of a scientific theory is its capacity to explain existing observations and generate predictions that can be experimentally tested and potentially refuted. The ICR framework fulfills this criterion by proposing seven biologically grounded predictions, each derived directly from one of the four functional phases described in Section 8. Together, the phases and predictions represent complementary views of the same model: mechanistic and experimental (**Table 2**).

**Table 2:** Testable predictions derived from the ICR theory.

Prediction	Type	Corresponding phase
Differential compartmental persistence during graft remodeling	Consistent	Selective epidermal elimination
Successful regeneration is preceded by a regulated immune transition	Consistent	Immune activation and cellular colonization
Preferential resolution of profibrotic pathways	Consistent	Immune activation and cellular colonization
Predominant colonization by recipient-derived cells	Discriminating	Regenerative remodeling
Preservation of native dermal architecture influences regenerative quality	Discriminating	Regenerative remodeling
Transient reactivation of developmental programs	Discriminating	Regenerative remodeling
Regulated alloimmunity is causally required for regeneration	Discriminating	All four phases

Seven predictions were classified as consistent or discriminating. Consistent: an observation that strengthens the theory without distinguishing it from the alternative explanations. Discriminating: a prediction whose failure would refute the theory.

A distinction should be made between consistent and discriminatory predictions. The former are compatible with ICR but may also be observed in other regenerative models; the latter test specific postulates of the theory and allow it to be distinguished from alternative hypotheses. Confirmation of a consistent prediction would strengthen the theory without proving it to be true. Conversely, the refutation of a discriminating prediction would require the revision of one of its central assumptions.

Prediction 1. Differential compartmental persistence during graft remodeling (consistent). Arising from the selective epidermal elimination phase, ICR proposes that the two compartments follow distinct biological fates during graft evolution. Therefore, the epidermis should disappear significantly earlier than the dermis, whereas the dermal matrix should transiently preserve its structural organization and continue to participate in tissue remodeling. This theory would be challenged if the epidermis and dermis were eliminated simultaneously or if they exhibited equivalent rates of disappearance.

Prediction 2. Successful regeneration is preceded by a regulated immune transition (consistent). Arising from the immune activation phase, this prediction holds that if the immune response actively organizes the regenerative process, a progressive transition should be observed from early inflammatory profiles to phenotypes associated with constructive remodeling, angiogenesis, and tissue repair. This theory would be challenged if regeneration occurred in the absence of such a transition or under sustained predominance of profibrotic signals.

Prediction 3. Regeneration is associated with the preferential resolution of profibrotic pathways (consistent). Additionally, arising from the immune activation phase and formulated as a direct contrast to pathological scarring discussed in Section 8.2, ICR predicts preferential resolution of TGF-β-dependent signaling, decreased myofibroblast differentiation, and a more physiological organization of collagen than in conventional fibrotic wound healing. The distinction is empirically decisive: an identical preserved matrix implanted in an environment in which immune resolution fails should yield fibrosis rather than regeneration. This theory would be challenged if the molecular profiles of the regenerated tissues were indistinguishable from those observed in fibrosis.

Prediction 4. The neodermis is predominantly colonized by recipient-derived cells (discriminating against the cellular survival model). Arising from the regenerative remodeling phase, this theory proposes that regeneration depends not on the prolonged survival of donor cells but on the instructive capacity of the preserved ECM. Therefore, the cellular populations within the neodermis should progressively be recipient-derived. This prediction distinguishes ICR from models that attribute regenerative effects to the functional persistence of donor cells and would be challenged if regeneration depended on the prolonged persistence of donor-derived cells.

Prediction 5. The preservation of native dermal architecture influences the quality of regeneration (discriminating against nonspecific repair models). In addition, arising from the regenerative remodeling phase, ICR assigns a central role to the information contained in the extracellular matrix. Matrices that better preserve the native dermal architecture should therefore promote more organized and less fibrotic responses than matrices with substantially altered architecture. This prediction discriminates ICR from models that regard the matrix as an undifferentiated physical support and would be challenged if regenerative quality proved independent of the degree of matrix preservation.

Prediction 6. Regeneration is accompanied by the transient reactivation of developmental programs (discriminating against the purely structural model). Completing the regenerative remodeling phase, the theory proposes that regeneration involves the temporary re-expression of molecular programs normally restricted to embryonic skin development or highly regenerative states, including developmental transcriptional signatures associated with embryonic skin morphogenesis and regenerative wound healing. This prediction distinguishes ICR from purely structural models because it implies an active biological response by the host rather than the passive use of a matrix scaffold, and would be challenged if the observed transcriptomic profiles were indistinguishable from those associated with conventional fibrotic repair.

Prediction 7. Regulated alloimmunity is causally required for regeneration (discriminating against both the purely structural and immune evasion models). The most specific prediction of ICR, which cuts across all four phases, is that regeneration does not depend solely on the presence of a preserved matrix. If the regulated alloimmune response is a causal component of this process, its experimental disruption should impair neodermis formation, even when the matrix architecture remains intact. This prediction distinguishes ICR from both purely structural models and models based solely on immune evasion, positioning immunity as a causal component, rather than a bystander. This theory would be challenged if the organized regeneration of a functional neodermis proceeded normally despite disruption of the proposed immunological mechanisms; a partial or disorganized response under such conditions would instead indicate that regulated alloimmunity optimizes rather than strictly enables the process.

Collectively, these predictions transform the ICR from a conceptual framework into a falsifiable biological model. Their systematic confirmation would progressively strengthen the theory, whereas failure of its discriminating predictions would require the revision of its central assumptions. Thus, the value of ICR does not reside in claiming certainty but in generating specific, experimentally testable hypotheses capable of advancing the understanding of cutaneous regeneration.

## 11 Conceptual and clinical implications of immunoguided cutaneous regeneration

If the theory proposed in this study is correct, its implications extend beyond the specific field of skin allografts and reach broader questions related to tissue immunology, regenerative medicine, and repair biology.

### 11.1 From rejection as failure to rejection as a biological transition

For much of the history of transplantation, immune rejection has been interpreted as an exclusively destructive phenomenon, the inevitable outcome of which is the functional graft loss. ICR does not challenge the immunological nature of these effects but rather proposes a reinterpretation of their biological effects. Under certain conditions, the elimination of graft cellular components may coexist with tissue repair and remodeling. This reinterpretation does not redefine rejection but rather the biological consequences that may follow under specific structural and immunological conditions. From this perspective, immune rejection and tissue regeneration are not necessarily mutually exclusive processes but may represent sequential components of a coordinated biological response. In this context, rejection ceases to represent merely the end of the graft’s biological history and instead becomes a potential transitional phase leading to subsequent tissue reorganization (**Table 3**).

**Table 3:** From the classical paradigm to immunoguided cutaneous regeneration.

Classical paradigm	ICR
Rejection = failure	Rejection = transition
Destructive immunity	Organizing immunity
Graft as tissue	Graft as microenvironment
Donor cells	Matrix + host
Temporary coverage	Regenerative platform

Synthetic contrast between the classical interpretation of the allograft and its reinterpretation under ICR.

### 11.2 The allograft as a transient instructive microenvironment

Classical transplantation practices have primarily valued grafts according to the survival of their cells and permanent integration into the recipient. ICR proposes a complementary perspective: the therapeutic value of certain allografts may reside less in cellular persistence and more in their capacity to transiently provide an instructive microenvironment capable of directing the recipient’s regenerative response.

### 11.3 Implications for the design of dermal substitutes

If the extracellular matrix is the principal instructive agent of the regenerative process, the criteria used to design dermal substitutes may need to be reconsidered. From this perspective, the central question shifts from how acellular or immunologically inert a material is to how much biological information it preserves and presents to the host. Three-dimensional architecture, matrix organization, and preservation of regulatory signals may become as important as, or even more important than, the simple reduction of antigenic load. From this perspective, the preservation of biological information becomes a design objective in itself, complementing rather than replacing current strategies aimed at minimizing immunogenicity.

### 11.4 Clinical implications for CTSA

ICR suggests that cryopreserved total skin allografts may play a broader role than temporary biological dressings (67). In addition to protecting the wound, a preserved full-thickness dermis may function as an instructive scaffold capable of promoting more organized dermal regeneration. If this interpretation is correct, the clinical evaluation of these grafts should incorporate variables related to the quality of the regenerated tissue, including dermal organization, vascularization, elasticity, functionality, and fibrosis, in addition to the duration of graft coverage. From this perspective, the living donor model acquires additional value by providing access to the human dermis with preserved architecture and high biological quality (64, 68). Consequently, future clinical studies should evaluate not only graft persistence but also the biological quality of the regenerated dermis as predicted by the ICR framework.

### 11.5 A possible bridge between transplantation and regenerative medicine

Historically, transplantation immunology and regenerative medicine have evolved as relatively independent disciplines. ICR suggests that the two may share common biological mechanisms. From this perspective, skin allotransplantation ceases to be solely a model of immune rejection and can also be understood as a model of tissue regeneration mediated by the interaction between immunity and extracellular matrix. Instead of representing separate biological disciplines, transplantation immunology and regenerative medicine may describe complementary aspects of the same host response to preserved extracellular matrices.

### 11.6 Beyond the skin

Although ICR arises from observations made in skin allografts, its principles may extend to other systems based on preserved extracellular matrix. This possibility remains to be demonstrated, but it raises the hypothesis that similar interactions between immunity and the extracellular matrix may participate in the regeneration of different tissues.

### 11.7 Translational challenges and limitations

The proposed framework describes favorable biological conditions in idealized terms; its clinical translation is subject to variables that may narrow or abolish the immunoregenerative window. Acknowledging these limitations does not weaken the theory but specifies the conditions under which it may be expected to operate.

The first is infection. Viable biological dressings have traditionally been regarded as superior to synthetic dressings in controlling wound bioburden, owing to their capacity for active cellular defense. However, CTSA occupies an intermediate position: terminal irradiation eliminates the bioburden of the graft itself but also its cellular viability, so that it lacks the active defensive mechanisms of a fresh allograft. A structurally preserved yet acellular dermis does not necessarily reproduce antimicrobial capacity, and its behavior toward microbial colonization or a pre-existing biofilm in the recipient bed remains to be characterized. This is relevant because an established infection would replace the regulated, self-limiting inflammation that the model requires with sustained, pathological-type inflammation, precisely the state that ICR identifies as incompatible with regeneration.

The second factor is the timing of implantation. The proposed sequence presupposes a recipient bed capable of mounting a coordinated immune response. Early versus delayed excision and the position of the ICR window relative to the acute inflammatory phase of the wound may determine whether the graft is implanted into a permissive environment or into one already dominated by profibrotic signaling.

The third is injury severity, which introduces a paradox that the model must address. In severely burned patients, systemic inflammatory response and subsequent immunoparalysis profoundly alter host immune reactivity. The same regulated alloimmune response that ICR posits as the driver of regeneration may be attenuated or disorganized in patients with the greatest body surface involvement. Therefore, the patient most likely to benefit from the graft may be the least able to sustain the proposed mechanism.

The fourth is extracellular matrix degradation. The instructive window depends on the dermal architecture and its regulatory components persisting long enough to direct the remodeling. In a metalloproteinase-rich microenvironment, degradation kinetics may shorten this window. To this is added the effect of irradiation itself: the 25 kGy dose that confers sterility and reduces immunogenicity may simultaneously modify collagen cross-linking and cleave chains, altering both biomechanical properties and matricryptic signaling. Therefore, the sterilization dose represents a balance between microbiological safety and the instructive integrity of the matrix, and its optimization remains an open experimental objective.

The fifth arises from the very feature that the model highlights as an advantage: preservation of full dermal thickness. A thicker graft imposes a greater revascularization demand than a split-thickness graft because the vascular front of the wound bed must traverse a greater distance to nourish the entire construct. The same property that endows the CTSA with its wealth of instructive information may therefore hinder or delay the initial engraftment. Thus, there is a trade-off between the amount of preserved dermal architecture and the ease of integration, the optimization of which remains an open clinical question. Nevertheless, the available clinical series documents that the CTSA engrafts and revascularizes despite its full thickness, suggesting that this revascularization demand constitutes a real but surmountable cost rather than an intrinsic impediment (27, 28, 64).

A further limitation is biological rather than translational and defines the ceiling of what the model can explain. The regeneration of cutaneous appendages depends not only on an instructive matrix but also on specialized immune cell populations. M2 macrophages contribute to hair follicle neogenesis by producing factors such as IGF-1 and FGF-2, whereas dermal γδ T cells promote this process through FGF9 secretion and activation of Wnt-associated pathways (69, 70). The relative scarcity of these populations in adult human skin may explain the exceptional nature of complete restoration of hair follicles and glands after injury (69). Consequently, ICR may explain the reconstruction of dermal architecture and reduction of fibrosis without necessarily implying complete regeneration of cutaneous appendages. Recognizing this limitation does not weaken the theory; rather, it precisely defines its domain of validity.

These considerations delimit the validity of the model. ICR does not hold that regeneration invariably follows graft application but that it emerges when a preserved dermal extracellular matrix and a regulated, resolving alloimmune response converge within a permissive biological context. Therefore, ICR describes a context-dependent regenerative process that emerges only when structural, immunological, and temporal conditions converge. Each of the variables above may prevent convergence, and their systematic study constitutes the necessary bridge between the conceptual framework proposed here and its eventual clinical validation. Collectively, these implications illustrate that ICR should not be viewed as a definitive explanation of cutaneous regeneration but as a framework for generating new biological questions that can be addressed experimentally.

## 12 Conclusions

The ICR is proposed as a biologically plausible and experimentally testable conceptual framework that explains observations that are not fully accounted for by the classical interpretation of skin allotransplantation. The theory suggests that, under specific conditions, the interaction between a preserved extracellular matrix and a regulated alloimmune response can generate a transient immunoregenerative microenvironment that supports tissue regeneration.

The model is based on a recurrent observation described throughout the history of skin allotransplantation: the progressive elimination of graft cellular components may coexist with the relative persistence of the dermis and its extracellular architecture. These phenomena are particularly evident in cryopreserved total skin allografts, where the preservation of full dermal thickness retains more native structural and biochemical information within the extracellular matrix than conventional split-thickness allografts. From this perspective, the observed regenerative capacity would depend less on the prolonged survival of donor cells than on the dynamic interaction between the preserved matrix and the recipient’s immune and regenerative responses.

ICR further proposes that CTSA may function as a platform capable of transiently recreating an immunoregenerative microenvironment, allowing coordinated interactions among immunity, the extracellular matrix, and recipient-derived cells. Under this interpretation, skin allotransplantation ceases to be solely a model of immune rejection and may also be understood as a potential model of tissue regeneration.

Nevertheless, ICR should be regarded as a biological hypothesis rather than as a proven mechanism. The evidence reviewed is consistent with the proposed model but does not establish a definitive causal relationship between the variables. Its principal strength lies in integrating previously dispersed historical, experimental, and clinical observations into a coherent explanatory framework and, above all, generating specific and potentially falsifiable predictions. Central to the model is the proposition that regeneration emerges when a preserved dermal extracellular matrix and a regulated, resolving alloimmune response converge within a permissive biological context, a context-dependent process rather than the consequence of any single factor.

If these predictions are confirmed, cutaneous regeneration may not be understood as a biological exception but as a latent capacity of the host that emerges when an instructive matrix converges with an appropriately regulated immune response. In this scenario, ICR provides a conceptual framework capable of bridging traditionally separate fields, including transplantation immunology, tissue engineering, and regenerative medicine. Whether future studies ultimately confirm or refine this framework, the ICR provides a structured conceptual foundation for investigating how immunity, extracellular matrix biology, and tissue architecture interact to determine the balance between fibrosis and regeneration.

## Data Availability

The original contributions presented in the study are included in the article/supplementary material, further inquiries can be directed to the corresponding author.
